# Comparison of Ambulatory Care Access and Quality for Beneficiaries With Disabilities Covered by Medicare Advantage vs Traditional Medicare Insurance

**DOI:** 10.1001/jamahealthforum.2021.4562

**Published:** 2022-01-14

**Authors:** Kenton J. Johnston, Hefei Wen, Harold A. Pollack

**Affiliations:** 1 Department of Health Management and Policy, Saint Louis University, St. Louis, Missouri; 2 Division of Health Policy and Insurance Research, Department of Population Medicine, Harvard Medical School & Harvard Pilgrim Health Care Institute, Boston, Massachusetts; 3 Crown Family School of Social Work, Policy, and Practice, University of Chicago, Chicago, Illinois; 4 Department of Public Health Sciences, University of Chicago, Chicago, Illinois; 5 Urban Health Lab, University of Chicago, Chicago, Illinois

## Abstract

**Importance:**

Medicare beneficiaries with disabilities aged 18 to 64 years face barriers accessing ambulatory care. Past studies comparing Medicare Advantage (MA) with traditional Medicare (TM) have not assessed how well these programs meet the needs of beneficiaries with disabilities.

**Objective:**

To compare differences in enrollment rates, ambulatory care access, and ambulatory care quality for beneficiaries with disabilities in MA vs TM.

**Design, Setting, and Participants:**

This cohort study included a nationally representative, weighted sample of 7201 person-years for beneficiaries aged 18 to 64 years with disability entitlement in the Medicare Current Beneficiary Survey from 2015 through 2018. Differences in program enrollment and in measures of access and quality by program enrollment were compared after adjusting for demographic, insurance, social, health, and area characteristics and after reweighting the sample by propensity to enroll in MA as estimated by observed confounders. Data analyses were conducted between November 1, 2020, and November 11, 2021.

**Exposures:**

Medicare Advantage vs TM program enrollment.

**Main Outcomes and Measures:**

Six patient-reported measures of ambulatory care access (usual source of care, primary care usual source of care, specialist visit) and quality (cholesterol screening, influenza vaccination, colon cancer screening).

**Results:**

The mean (SD) age of the overall study population was 52.1 (11.0) years; 49.5% were female and 50.5% were male; 1.6% were Asian/Pacific Islander; 17.4%, Black; 10.2% Hispanic; 1.4%, Native American; 65.1%, White, and 4.2%, multiracial. Among all beneficiaries living in the community, individuals with disability entitlement were less likely to enroll in MA than other beneficiaries (34.8% vs 41.2%). The final sample of beneficiaries with disabilities included 2444 person-years in MA and 4757 person-years in TM. Beneficiaries with disabilities in MA vs TM were more likely to be of a minority race or ethnicity (35.7% vs 27.6%) and less likely to be enrolled in private insurance (11.9% vs 25.0%). Comparing MA with TM among beneficiaries with disabilities, those in MA had significantly better rates of access to a usual source of care (90.2% vs 84.9%; adjusted propensity-weighted marginal difference [APWMD], 2.9%; 95% CI, 0.2%-5.7%), access to specialist visits (53.2% vs 44.8%; APWMD, 5.5%; 95% CI, 0.6%-10.5%), cholesterol screenings (91.1% vs 86.4%; APWMD, 3.8%; 95% CI, 0.9%-6.7%), influenza vaccinations (61.4% vs 51.5%; APWMD, 10.4%; 95% CI, 5.3%-15.5%), and colon cancer screenings (68.4% vs 54.6%; APWMD, 10.3%; 95% CI, 4.8%-15.8%).

**Conclusions and Relevance:**

In this cohort study, Medicare beneficiaries with disabilities were enrolled in MA at significantly lower rates than those without disabilities. However, MA was associated with significantly better ambulatory care access and quality for these beneficiaries on 5 of 6 measures compared with TM.

## Key Points

**Question:**

Do Medicare beneficiaries aged 18 to 64 years with disability entitlement have different rates of enrollment in Medicare Advantage (MA) vs traditional Medicare (TM) compared with other beneficiaries, and how do the 2 programs compare on rates of ambulatory care access and quality for beneficiaries with disabilities?

**Findings:**

In this cohort study of a nationally representative sample of 7201 person-years for Medicare beneficiaries in 2015 through 2018, beneficiaries with disability entitlement were significantly less likely to enroll in MA compared with those without disability entitlement. However, enrollment in MA vs TM was associated with better outcomes on 2 of 3 access measures and 3 of 3 quality measures for beneficiaries with disabilities.

**Meaning:**

Although Medicare beneficiaries with disabilities enrolled in MA at lower rates than other beneficiaries in this study, MA appeared to compare favorably with TM in meeting key ambulatory care access and quality measures for beneficiaries with disabilities.

## Teaser

This cohort study uses data from the Medicare Current Beneficiary Survey to compare differences in enrollment rates, ambulatory care access, and ambulatory care quality among beneficiaries with disabilities who are enrolled in Medicare Advantage vs traditional Medicare.

## Introduction

Medicare beneficiaries with disabilities aged 18 to 64 years comprise 15% of the Medicare population and experience considerable disparities in access to care compared with beneficiaries aged 65 years or older.^1,2^ However, access to care for this vulnerable population has been understudied. Medicare beneficiaries with disabilities may enroll in the traditional Medicare (TM) program administered by the Centers for Medicare & Medicaid Services (CMS) or in private Medicare Advantage (MA) plans.

Medicare beneficiaries with disabilities report greater difficulty accessing care and are less likely to receive needed preventive care than are other beneficiaries.^3,4,5,6,7,8^ Traditional Medicare beneficiaries with disabilities aged 21 to 64 years are 33% and 49% less likely to have access to primary care clinicians (PCCs) and specialists, respectively, than are beneficiaries without disabilities.^2^ Lack of access to high-quality ambulatory care is associated with negative clinical outcomes.^2,9,10,11^

Comparisons of MA with TM in the general population indicate that MA beneficiaries have lower use of outpatient visits^12,13,14^ yet receive similar or better preventive care.^12,15,16,17,18,19^ In prior years, sicker beneficiaries were more likely to select into TM vs MA,^20,21,22^ although in recent years, this may no longer be the case as younger beneficiaries with disabilities enroll in MA in higher numbers.^23,24^ The consequences of MA vs TM for risk selection, access to care, and quality of care for beneficiaries with disabilities are unknown.

Beneficiaries with disabilities may also enroll in MA special needs plans (SNPs) designed to coordinate and integrate care for dual Medicaid enrollees or beneficiaries with chronic and disabling conditions.^25,26^ However, it is unclear whether SNPs consistently provide high-quality, integrated, cost-efficient care as intended.^26,27,28^ To our knowledge, only 1 study has assessed SNPs among beneficiaries aged 18 to 64 years, and it found that dual beneficiaries who enrolled in SNPs vs other plans had higher hospitalization rates.^29^

The present study focused on 2 primary questions: (1) Do beneficiaries with disabilities enroll in MA at different rates than beneficiaries without disabilities? (2) How do rates of ambulatory care access and quality for beneficiaries with disabilities compare in MA vs TM? In secondary exploratory analyses, we expanded these questions to compare beneficiaries in MA SNPs vs non-SNPs vs TM.

## Methods

This cohort study was deemed exempt by the Saint Louis University Institutional Review Board, and informed consent was waived for the use of deidentified data. The study followed Strengthening the Reporting of Observational Studies in Epidemiology (STROBE) reporting guidelines for cohort studies.^30^

### Data and Study Population

A cohort study was conducted using MA and TM beneficiaries in the Medicare Current Beneficiary Survey (MCBS) for the years 2015 through 2018. The MCBS is an annual, nationally representative sample of the Medicare population with a rotating cohort design.^31^

To calculate annual MA enrollment rates, a sample was created of all Medicare beneficiaries with and without current disability entitlement and at least 1 month of Part A and B enrollment living in the community. To compare MA with TM among beneficiaries with disabilities, the sample was limited to beneficiaries aged 18 to 64 years with current disability entitlement, at least 12 months of exclusive Part A and B enrollment in MA or TM, completion of the MCBS, and residence in a US zip code. The MCBS cross-sectional survey weights were used to compute nationally representative estimates. These weights account for the selection probability of each person sampled and include adjustments for stratified sampling design and survey nonresponse.^31^

### Exposure and Outcome Variables

The primary exposure variable, MA vs TM enrollment, was identified using Medicare administrative data linked to the MCBS. The secondary exposure variable, SNP vs non-SNP enrollment, was identified for the 89% of MA beneficiaries who had MA plan and contract identifiers in the MCBS that could be linked to published SNP data.^32^ The outcome variables were 6 measures of ambulatory care access and quality self-reported by beneficiaries. Some questions were asked in the following year in reference to the previous year, and respondents who dropped out of the MCBS at the end of the previous year were excluded.

Three measures of ambulatory care access were assessed. (1) Access to a usual source of care (USOC) was identified as beneficiaries who self-reported that they went to a particular place for medical care and identified the place as a doctor’s office, clinic, health center, or home. (2) Access to a USOC that was a PCC was identified as beneficiaries who reported the specialty of their USOC clinician to be family practice, general practice, geriatrics, gynecology, internal medicine, osteopathy, or physician assistant. (3) Access to a specialist visit in the past year was identified as beneficiaries who reported seeing a specialist apart from a PCC.

Three preventive care services received in the ambulatory setting were assessed as quality measures: (1) blood cholesterol checks in the past year for beneficiaries with diabetes, ischemic heart disease, or heart failure, indicating they were at high risk for cardiovascular complications^33^; (2) annual influenza vaccinations for all beneficiaries^34^; and (3) colon cancer screening for beneficiaries aged 45 years or older who self-reported not having colon cancer and who received within the past 5 years either a colonoscopy or sigmoidoscopy in the doctor’s office or a fecal occult blood test at home or in the doctor’s office.^35^

### Beneficiary Characteristics

Age and sex were assessed using administrative records, and veteran status was self-reported. Beneficiaries self-reported their own race and ethnicity in the MCBS. The National Institute on Minority Health and Health Disparities racial and ethnic framework^36^ was used to identify beneficiaries as belonging to a racial or ethnic minority group if they reported a race or ethnicity of Asian/Pacific Islander, Black, Hispanic, or Native American. We identified beneficiaries who reported they were White or multiracial and not Hispanic as being of other race and ethnicity. We assessed race and ethnicity because racial and ethnic minority beneficiaries experience disparities in access to care in both MA and TM.^19^

We used administrative records to identify whether beneficiaries had other forms of health insurance that may influence their access to and quality of care apart from MA vs TM enrollment, including Medicaid, private insurance, and Part D (drug) insurance.

Social risk factors were assessed using patient-reported measures of material capital (annual income and federal poverty status), human capital (highest level of education attained), and social support (living alone).

Health behaviors and status were assessed using patient-reported measures of tobacco use, alcohol abuse (at least 4 drinks most days), and obesity (based on height and weight). Poor self-rated health was identified for those who rated their health in the past year as fair or poor. Functional impairment was measured as counts (ranging from 0 to 6 functional limitations) of self-reported difficulty with activities of daily living (ADLs) and instrumental ADLs (IADLs).

Four common chronic conditions in Medicare populations—diabetes, heart failure, ischemic heart disease, and asthma/chronic obstructive pulmonary disease—were identified if beneficiaries reported ever having those conditions. Mental health conditions were identified for those who reported any psychiatric illness, including depression. Finally, the presence of an intellectual and/or developmental disability (IDD) was identified for those who reported (or their proxy respondent reported) an IDD.

Local area variables were assessed as (1) MA market penetration (ie, uptake) rate in beneficiaries’ counties of residence, identified using CMS data^37^; and (2) rural vs urban residence, identified by linking beneficiaries’ residential zip codes to rural-urban commuting area codes.^38^

### Propensity Score Adjustment

The sample was reweighted by propensity to enroll in MA as estimated by observed confounders. This adjustment was done to change the distribution of confounders in both MA and TM beneficiaries so that they were the same as the distribution in the national Medicare population with disability entitlement (eMethods and eTables 1-2 in the Supplement).

### Statistical Analysis

Data analyses were conducted between November 1, 2020, and November 9, 2021. Unadjusted rates of enrollment in MA, and in SNPs vs non-SNPs, among beneficiaries with vs without disabilities were compared and then adjusted for county-level MA market penetration and state fixed effects.

Descriptive statistics (with and without propensity weight adjustment) were computed on beneficiaries’ characteristics, and the Wald test was used to compare differences by beneficiaries’ MA vs TM enrollment status.

Unadjusted rates of the 6 outcomes were computed for beneficiaries with disabilities enrolled in MA vs TM, as well as for those in MA SNPs vs MA non-SNPs vs TM.

Multivariable logistic regression models with and without propensity weight adjustment were estimated at the person-year level to assess the association between MA (vs TM) enrollment and the 6 outcomes. Models were adjusted for the beneficiary characteristics listed above as well as for county-level MA market penetration rates and state fixed effects. All models included year fixed effects to control for secular trend and were adjusted for the complex survey design of the MCBS and clustered standard errors on beneficiaries to adjust for intraperson correlation over time. Results were reported as marginal differences, which can be interpreted as percentage point differences in the outcomes associated with the exposure variables. The propensity-weighted results can be interpreted as the average national treatment effect of MA vs TM on the 6 outcomes among beneficiaries with disabilities.

In secondary exploratory analyses, the above multivariable regression models were re-estimated with enrollment in MA SNPs vs TM and in MA non-SNPs vs TM as the exposures. This analysis was done to assess whether there was a different pattern of associations with each of the 6 outcomes for MA beneficiaries in SNPs vs non-SNPs.

In sensitivity analyses, the primary analytic models were re-estimated after (1) using fixed effects for the Dartmouth hospital referral regions of beneficiaries’ residences instead of states, (2) relaxing continuous enrollment criteria and including beneficiaries with less than 12 months of enrollment, and (3) interacting counts of ADL and IADL limitations with MA enrollment to test for heterogeneous treatment associations by level of functional impairment.

The threshold for statistical significance was *P* < .05 with 2-sided tests. Analyses were performed using SAS, version 9.4 (SAS Institute Inc) and Stata, version 16 (StataCorp LLC).

## Results

### Description of Study Population and MA Enrollment Rates

The mean (SD) age of the overall study population was 52.1 (11.0) years; 49.5% were female and 50.5% were male; 1.6% were Asian/Pacific Islander; 17.4%, Black; 10.2% Hispanic; 1.4%, Native American; 65.1%, White, and 4.2%, multiracial. Of 52 587 (unweighted) person-years in the community with at least 1 month of Part A and Part B enrollment, 21 144 (40.2%) had exclusive enrollment in MA and 28 984 (55.1%) had exclusive enrollment in TM (eFigure in the Supplement). Of those exclusively enrolled in MA and TM, there were 2713 and 5206 person-years with disability entitlement, respectively. After excluding those who lacked 12-month enrollment or had missing data, the final sample consisted of 7201 person-years, including 2444 person-years in MA (weighted 9 583 292) and 4757 person-years in TM (weighted 15 412 888). Beneficiaries excluded from the study had less enrollment and were more likely to die (eTable 3 in the Supplement); however, the mortality rate was not statistically different for MA vs TM beneficiaries (eTable 4 in the Supplement).

Beneficiaries with disabilities were less likely to enroll in MA than those without disabilities (34.8% vs 41.2%; adjusted difference [AD], −6.7%; 95% CI, –4.3% to –9.1%) (Table 1). Among MA beneficiaries, individuals with vs without disabilities were more likely to enroll in SNPs (28.1% vs 8.7%; AD, 13.0%; 95% CI, 10.0% to 16.1%).

**Table 1.** Weighted Percentages of Medicare Beneficiaries With Exclusive Annual Enrollment in Medicare Advantage (MA) by Disability Entitlement, 2015-2018

Variable	Disability entitlement	No disability entitlement	% (95% CI)	
			Unadjusted difference	Market-adjusted difference^a^
Total unweighted patient-years, No.^b^	8716	43 871	NA	NA
Exclusive MA enrollment among all beneficiaries, %	34.8	41.2	–6.4 (–9.0 to –3.8)	–6.7 (–9.1 to –4.3)
SNP enrollment among MA beneficiaries^c^				
SNP (as % of MA beneficiaries)^d^	28.1	8.7	19.4 (15.3 to 23.4)	13.0 (10.0 to 16.1)
Non-SNP (as % of MA beneficiaries)	71.9	91.3	NA	NA

Abbreviations: NA, not applicable; SNP, special needs plan.

^a^ We adjusted for county-level MA market penetration rate (proportion of Medicare beneficiaries in the beneficiary's county enrolled in MA) and state fixed effects to adjust for state policy differences and state differences in supply of medical services, clinician practice intensity, and coding intensity. We included year fixed effects to adjust for secular trend.

^b^ Medicare beneficiaries living in the community with at least 1 month of enrollment in Part A and B benefits. Unweighted sample sizes are reported. Estimates from the 2015-2018 Medicare Current Beneficiary Survey were weighted to be nationally representative using cross-sectional weights accounting for the overall annual selection probability of each person sampled and including adjustments for the stratified sampling design, survey nonresponse, and coverage error.

^c^ Sampling MA beneficiaries with an available MA plan and contract identification numbers in the Medicare Current Beneficiary Survey linked to public data on MA special needs plans published annually by the Centers for Medicare & Medicaid Services at the plan and contract level (n = 2368 of 2732 for beneficiaries with disability entitlement and n = 16 408 of 18 412 for beneficiaries without disability entitlement).

^d^ SNPs for chronic and disabling conditions, dually enrolled Medicare-Medicaid beneficiaries, and beneficiaries institutionalized in long-term care.

Beneficiaries with disabilities in MA were more likely to belong to a racial or ethnic minority group (35.7% vs 27.6%), less likely to be enrolled in private insurance (11.9% vs 25.0%), more likely to be enrolled in Part D (97.8% vs 80.3%), and less likely to live in poverty (37.7% vs 43.7%) than beneficiaries with disabilities in TM (Table 2). Beneficiaries with disabilities in MA were more likely to have diabetes (40.5% vs 34.4%) and ischemic heart disease (18.8% vs 14.2%), less likely to have an IDD (13.6% vs 16.5%), less likely to live in rural areas (17.1% vs 28.9%), and more likely to live in counties with higher MA penetration rates (37.7% vs 29.3%) than those in TM.

**Table 2.** Characteristics of Community-Dwelling Medicare Beneficiaries With Disability Entitlement Enrolled in Medicare Advantage (MA) vs Traditional Medicare (TM) Insurance, Aged 18-64 Years, 2015-2018

Characteristic	Unadjusted weighted %		*P* value^a^	Propensity-adjusted weighted %		*P* value^a^
	MA	TM		MA	TM	
Total number of patient years, unweighted, No.	2444	4757	NA	2444	4757	NA
Demographic characteristics						
Age, mean (SD), y	54.0 (9.2)	50.8 (11.9)	<.001	52.0 (10.0)	51.9 (10.3)	.88
Sex						
Male	48.2	51.9	.09	52.7	50.0	.38
Female	51.8	48.1		47.3	50.0	
Minority race and ethnicity	35.7	27.6	<.001	30.6	30.9	.23
Asian/Pacific Islander	1.0	2.0		0.8	1.9	
Black	19.3	16.2		18.5	18.1	
Hispanic	14.2	7.8		10.0	9.5	
Native American	1.1	1.6		1.3	1.5	
White	59.9	68.3		65.6	65.3	
Multiracial	4.4	4.0		3.9	3.8	
Veteran (served in US armed forces)	6.2	8.7	.05	7.0	7.5	.75
Other health insurance						
Medicaid (dually enrolled)	51.7	55.4	.13	53.2	55.7	.43
Private (including medical, drug, vision, and dental)	11.9	25.0	<.001	22.2	21.3	.79
Medicare Part D (stand-alone or with Part C)	97.8	80.3	<.001	86.0	87.0	.78
Social risk factors						
Annual income in thousands, mean (SD)	24.6 (35.6)	25.7 (32.6)	.45	25.1 (32.1)	25.6 (29.8)	.78
Poverty (≤100% of federal poverty level)	37.7	43.7	.003	39.7	42.1	.36
Education						
No high school or college education	21.5	22.2	.59	21.2	22.6	.46
High school/some college education	69.0	69.4		68.9	69.5	
College/graduate school education	9.5	8.3		10.0	7.9	
Lives alone	30.2	27.2	.12	30.8	29.0	.43
Health behaviors and status						
Current smoker	32.1	33.3	.57	34.9	32.5	.32
Alcohol abuse (≥4 alcoholic drinks most days)	20.9	18.9	.26	21.3	20.2	.75
Obesity (BMI ≥30)	45.4	46.5	.62	47.2	46.9	.94
Poor self-rated health	56.5	56.3	.91	53.9	56.7	.27
Activities of daily living with difficulty/cannot do (0-6), mean (SD)	1.4 (1.7)	1.4 (1.9)	.50	1.4 (1.7)	1.4 (1.7)	.93
Instrumental activities of daily living with difficulty/cannot do (0-6), mean (SD)	1.7 (1.6)	1.8 (1.9)	.02	1.7 (1.7)	1.8 (1.7)	.16
Health conditions						
Diabetes	40.5	34.4	.004	34.2	36.6	.41
Heart failure	9.9	8.7	.37	8.4	9.0	.64
Ischemic heart disease	18.8	14.2	.003	16.1	15.4	.74
Chronic obstructive pulmonary disease/asthma	33.8	31.7	.27	31.6	33.1	.55
Mental health condition^b^	63.8	65.7	.34	62.5	65.0	.32
Intellectual and/or developmental disability	13.6	16.5	.02	15.1	14.7	.82
Local area characteristics						
Rural	17.1	28.9	<.001	22.2	24.1	.56
Urban	82.9	71.1		77.8	75.9	
MA market penetration rate	37.7	29.3	<.001	33.4	32.8	.49

Abbreviations: BMI, body mass index (calculated as weight in kilograms divided by height in meters squared).

^a^ *P* value on the Wald test of significance, equivalent to the *F* statistic for continuous variables and the χ^2^ statistic for categorical variables.

^b^ Self-reported any psychiatric illness, including depression.

Covariates were balanced after propensity-weighting the sample, with no remaining significant differences on any beneficiary characteristics (Table 2).

Medicare Advantage beneficiaries with disabilities in SNPs vs non-SNPs were more likely to belong to racial or ethnic minority groups, be dually enrolled in Medicaid, live in poverty, lack a high school education, smoke, have diabetes, and have an IDD (eTable 5 in the Supplement).

### Association of MA vs TM Enrollment With Access and Quality Outcomes

Comparing MA vs TM among beneficiaries with disabilities, those in MA displayed significantly better rates of access to a USOC (90.2% vs 84.9%; adjusted propensity-weighted marginal difference [APWMD], 2.9%; 95% CI, 0.2%-5.7%), access to specialist visits (53.2% vs 44.8%; APWMD, 5.5%; 95% CI, 0.6%-10.5%), cholesterol screenings (91.1% vs 86.4%; APWMD, 3.8%; 95% CI, 0.9%-6.7%), influenza vaccinations (61.4% vs 51.5%; APWMD, 10.4%; 95% CI, 5.3%-15.5%), and colon cancer screenings (68.4% vs 54.6%; APWMD, 10.3%; 95% CI, 4.8%-15.8%) (Table 3). Marginal differences of MA vs TM were statistically similar for propensity-weighted and non-propensity-weighted results.

**Table 3.** Association of Medicare Advantage (MA) vs Traditional Medicare (TM) With Ambulatory Care Access and Quality for Beneficiaries With Disability Entitlement, 2015-2018

Variable	Unadjusted results			Adjusted marginal difference of MA vs TM, (95% CI)	
	MA	TM	Absolute difference (95% CI)	Regression results^a^	Propensity-weighted regression results^b^
Access^c^					
Usual source of care, %	90.2	84.9	5.3 (3.2 to 7.4)	3.7 (1.5 to 5.9)	2.9 (0.2 to 5.7)
Usual source of care is PCC, %	77.4	70.1	7.2 (3.5 to 11.0)	5.0 (1.1 to 9.0)	3.0 (– 0.8 to 6.8)
Specialist visit, %	53.2	44.8	8.3 (4.3 to 12.3)	4.9 (0.7 to 9.0)	5.5 (0.6 to 10.5)
Quality					
Annual cholesterol screen, %^d^	91.1	86.4	4.7 (1.7 to 7.8)	3.5 (0.8 to 6.2)	3.8 (0.9 to 6.7)
Annual flu shot, %^e^	61.4	51.5	9.9 (6.0 to 13.8)	10.1 (5.3 to 14.8)	10.4 (5.3 to 15.5)
Colon cancer screening, %^f^	68.4	54.6	13.8 (9.3 to 18.3)	11.5 (6.4 to 16.5)	10.3 (4.8 to 15.8)

Abbreviation: PCC, primary care clinician.

^a^ We estimated multivariable logistic regression models for each outcome that also adjusted for the characteristics listed in Table 2 (with race and ethnicity collapsed into minority vs other). We added fixed effects for the states that beneficiaries resided in to control for state policy differences and state differences in supply of medical services, clinician practice intensity, and coding intensity. We included year fixed effects to control for secular trend and adjusted our *P* values for the complex survey design of the Medicare Current Beneficiary Survey and intra-person correlation over time. We used Stata's Margins command to report our results as the marginal difference of MA vs TM for the dependent variables by modeling the response in the dependent variables to the exposure variable at the population means.

^b^ We estimated the same multivariable logistic regression models as in a, but this time reweighting the sample using the propensity score weights described previously to change the distribution of observed confounders in both the treated (MA) and untreated (TM) beneficiaries so that they are the same as the distribution in the entire sample. These estimates should be interpreted as what we would expect to see if every Medicare beneficiary in our nationally representative sample enrolled in MA vs what we would expect to see if nobody enrolled in MA (ie, the average treatment effects).

^c^ Unweighted sample n = 6525. Met baseline study inclusion and responded to Medicare Current Beneficiary Survey questions for outcome variables.

^d^ Unweighted sample n = 2715. Met baseline study inclusion and exclusion criteria and self-reported having diabetes, ischemic heart disease, or heart failure and responded to Medicare Current Beneficiary Survey questions for outcome variable.

^e^ Unweighted sample n = 6462. Met baseline study inclusion and exclusion criteria and responded to Medicare Current Beneficiary Survey question for outcome variable.

^f^ Fecal occult blood test at home or physician’s office or colonoscopy or sigmoidoscopy within past 5 years, excluding patients who self-reported having colon cancer or were younger than 45 years. Unweighted sample n = 3233 for patients who met above criteria as well as baseline study inclusion and exclusion criteria and responded to Medicare Current Beneficiary Survey questions for outcome variable.

### Association of MA SNP and Non-SNP vs TM Enrollment With Access and Quality Outcomes

Comparing MA SNPs with TM, beneficiaries in MA SNPs displayed significantly better rates of influenza vaccinations (AD, 7.9%; 95% CI, 0.2%-15.6%), and colon cancer screenings (AD, 13.1%; 95% CI, 5.6%-20.5%) (Table 4). Comparing MA non-SNPs vs TM, those in MA non-SNPs displayed significantly better rates of access to a USOC (AD, 5.5%; 95% CI, 3.3%-7.7%), access to a primary-care USOC (AD, 9.3%; 95% CI, 4.5%-14.0%), access to specialist visits (AD, 5.9%; 95% CI, 1.3%-10.5%), cholesterol screenings (AD, 2.8%; 95% CI, 0.2%-5.4%), influenza vaccinations (AD, 11.3%; 95% CI, 5.0%-17.6%), and colon cancer screenings (AD, 11.4%; 95% CI, 5.5%-17.4%).

**Table 4.** Association of Medicare Advantage Special Needs Plans and Non–Special Needs Plans vs Traditional Medicare With Ambulatory Care Access and Quality for Beneficiaries With Disability Entitlement, 2015-2018

Variable	Unadjusted results, weighted %				Adjusted marginal difference (95% CI)^a^	
	Special needs plan	Non–special needs plan	Traditional Medicare	*P* value^b^	Special needs plan	Non–special needs plan
Access^c^						
Usual source of care	86.7	92.3	84.9	<.001	1.1 (–2.7 to 4.9)	5.5 (3.3 to 7.7)
Usual source of care is PCC	69.4	81.6	70.1	<.001	–0.7 (–5.9 to 4.4)	9.3 (4.5 to 14.0)
Specialist visit	41.8	57.7	44.8	<.001	0.0 (–7.8 to 7.8)	5.9 (1.3 to 10.5)
Quality						
Annual cholesterol screen^d^	88.3	91.7	87.1	.08	1.3 (–2.3 to 4.9)	2.8 (0.2 to 5.4)
Annual flu shot^e^	57.2	63.5	51.1	<.001	7.9 (0.2 to 15.6)	11.3 (5.0 to 17.6)
Colon cancer screening^f^	66.9	69.3	54.4	<.001	13.1 (5.6 to 20.5)	11.4 (5.5 to 17.4)

Abbreviation: PCC, primary care clinician.

^a^ We estimated multivariable logistic regression models for each outcome that also adjusted for the characteristics listed in Table 2 (with race and ethnicity collapsed into minority vs other). We added fixed effects for the states that beneficiaries resided in to control for state policy differences and state differences in supply of medical services, clinician practice intensity, and coding intensity. We included year fixed effects to control for secular trend and adjusted our *P* values for the complex survey design of the Medicare Current Beneficiary Survey and intra-person correlation over time. We used Stata's Margins command to report our results as the marginal difference of Medicare Advantage vs traditional Medicare for the dependent variables by modeling the response in the dependent variables to the exposure variable at the population means.

^b^ On a χ^2^ test for difference in proportions across Medicare Advantage Special Needs Plan vs Medicare Advantage non–special needs plan vs traditional Medicare.

^c^ Unweighted sample n = 6257. Met baseline study inclusion and responded to Medicare Current Beneficiary Survey questions for outcome variables.

^d^ Unweighted sample n = 2388. Met baseline study inclusion and exclusion criteria and self-reported having diabetes, ischemic heart disease, or heart failure and responded to Medicare Current Beneficiary Survey questions for outcome variable.

^e^ Unweighted sample n = 6019. Met baseline study inclusion and exclusion criteria and responded to Medicare Current Beneficiary Survey question for outcome variable.

^f^ Fecal occult blood test at home or physician’s office or colonoscopy or sigmoidoscopy within past 5 years, excluding patients who self-reported having colon cancer or were younger than 45 years. Unweighted sample n = 2840 for patients who met above criteria as well as baseline study inclusion and exclusion criteria and responded to Medicare Current Beneficiary Survey questions for outcome variable.

In sensitivity analyses, results were statistically similar under alternative specifications (eTables 6-7 in the Supplement). No heterogenous treatment effects by ADL and IADL limitations were found on 11 of 12 tests (eTable 8 in the Supplement).

## Discussion

In this nationally representative cohort study, Medicare beneficiaries with disabilities were enrolled in MA at significantly lower rates than their peers without disabilities. In addition, although there were some significant differences in beneficiary characteristics between MA and TM, we found no systematic evidence that MA served a more favorable risk profile of beneficiaries with disabilities than TM. Beneficiaries with disabilities who enrolled in MA did display better access to ambulatory care and appeared to receive better quality of care than their similar peers in TM. These results were statistically similar across unadjusted, adjusted, and propensity-weighted estimates (although adjusted and propensity-weighted point estimates were smaller), including adjustment for other insurance benefits, as well as state and market differences. Although the beneficial association of MA vs TM extended to both MA SNP and non-SNP beneficiaries on 2 of 3 quality outcomes, only MA non-SNP beneficiaries displayed significantly better rates than TM beneficiaries on all 6 access and quality outcomes.

These findings suggest that MA compares favorably with TM in meeting key preventive and ambulatory care needs of beneficiaries with disabilities. These findings also raise questions as to why MA enrollment rates are lower among beneficiaries with disabilities. Prior studies have found favorable selection into MA; sicker beneficiaries have higher rates of disenrollment from MA and switching to TM than their peers,^20,21,39^ although this may no longer be the case in recent years.^23^ Thus, it is possible that MA plans select against beneficiaries with disabilities. However, the present findings were robust to adjustment for MA county penetration and state and market differences, implying that lower MA enrollment rates among beneficiaries with disabilities are unlikely to reflect strategic decisions by MA plans to avoid areas with large populations of people with disabilities. Beneficiaries with disabilities may be less familiar with MA plan offerings than are elderly beneficiaries who receive regular MA marketing from sources they trust, such as the American Association of Retired Persons. More research is needed to understand why beneficiaries with disabilities are less likely to enroll in MA plans.

This study expands on prior findings in the general Medicare population that beneficiaries in MA appear to receive better preventive care for processes such as cancer screening and flu shots compared with the subpopulation of TM beneficiaries with disabilities who are younger than 65 years.^15,16,18,19,40^ This finding may be due to greater use and tracking of Healthcare Effectiveness Data and Information Set quality measures in MA plans that affect MA performance ratings.^16^ However, in contrast to prior studies of the general population finding lower use of outpatient visits in MA than TM,^12,13^ this study found that beneficiaries with disabilities in MA were more likely to have access to usual care and specialists in the ambulatory setting. This finding is important because beneficiaries with disabilities in TM are less likely to have access to PCCs and specialists on an annual basis than their peers without disabilities.^2^ By early 2020, 77% of MA beneficiaries also belonged to plans with telehealth benefits.^41^ It is possible that greater flexibility by MA plans in paying for home and telemedicine visits contribute to improved access for beneficiaries with disabilities.

The most surprising finding of this study was that the beneficial association of MA enrollment with access to care was concentrated among beneficiaries not enrolled in SNPs. The Medicare Payment Advisory Commission recently reported that only 18% of SNPs for dual beneficiaries have meaningful integration with Medicaid.^26^ Most beneficiaries with disabilities enrolled in SNPs in the present study were dual beneficiaries. More research is needed to understand whether MA SNPs are meeting the needs of beneficiaries with disabilities.

### Limitations

This study has several limitations. First, Medicare beneficiaries aged 18 to 64 years with disabilities were identified based on their current disability entitlement status in the Medicare program. This population did not include beneficiaries aged 65 or older who originally qualified for Medicare because of disability; nor did it include those aged 18 to 64 years with disabilities who have not qualified for Medicare.

Second, this was an observational study. The comparisons between MA and TM may be influenced by unobserved variables associated with plan or program enrollment. In comparative studies of MA vs TM, the confounding issues of primary concern include administrative coding bias due to diagnosis upcoding in MA,^42^ spillover effects of MA onto TM practice patterns within local areas,^43^ strategic MA plan entry and exit in local markets,^41^ beneficiary selection into MA vs TM based on health status, availability of other supplemental health insurance, and other factors.^44^ In this study, rich patient-reported data supplemented by administrative data were used to control for administrative coding bias (via patient-reported health instead of coded diagnoses), spillover effects (via adjustment for county MA penetration rates), regional differences (via state and hospital market fixed effects), and other beneficiary health insurance, including Medicaid, private insurance, and Part D, that may influence MA vs TM enrollment decisions. Although this study controlled for the most likely confounders, potential confounding due to other unobserved factors cannot be ruled out, and therefore the study estimates do not imply causality.

## Conclusions

In this nationally representative cohort study of Medicare beneficiaries covering the period 2015 through 2018, beneficiaries aged 18 to 64 years with disabilities were significantly less likely to enroll in MA than other beneficiaries. However, MA enrollment was significantly associated with better outcomes for ambulatory care access and quality on 5 of 6 measures among beneficiaries with disabilities.

## Notes

Supplement. **eMethods.** Propensity Score Weight Adjustment **eTable 1.** List of Covariates Used in Estimation of Propensity Score Adjustment **eTable 2.** Hosmer-Lemeshow Goodness of Fit Test for Propensity Score **eFigure.** Sample Selection Flowchart **eTable 3.** Comparing Medicare Beneficiaries with Disabilities Included Versus Excluded From Study Sample, Ages 18-64, 2015-2018 **eTable 4.** Characteristics of Community-Dwelling Medicare Beneficiaries with Disability Entitlement Enrolled in Medicare Advantage Versus Traditional Medicare Insurance, Ages 18-64, 2015-2018. After Relaxing Continuous Enrollment Inclusion Criteria **eTable 5.** Characteristics of Community-Dwelling Medicare Advantage Beneficiaries with Disability Entitlement by Special Needs Plan vs Non Special Needs Plan Enrollment, Ages 18-64, 2015-2018 **eTable 6.** Association of Medicare Advantage vs. Traditional Medicare with Ambulatory Care Access and Quality for Beneficiaries with Disability Entitlement, 2015-2018. With Dartmouth Hospital Referral Region Fixed Effects **eTable 7.** Association of Medicare Advantage vs. Traditional Medicare with Ambulatory Care Access and Quality for Beneficiaries with Disability Entitlement, 2015-2018. After Relaxing Continuous Enrollment Inclusion Criteria **eTable 8.** Association of Medicare Advantage vs. Traditional Medicare with Ambulatory Care Access and Quality for Beneficiaries with Disability Entitlement, 2015-2018. Testing for Heterogeneous Treatment Effects by Level of Functional Impairment
