# Supplementary material for: Comparison of Ambulatory Care Access and Quality for Beneficiaries With Disabilities Covered by Medicare Advantage vs Traditional Medicare Insurance
Source: JAMA Health Forum. 2022 Jan 14;3(1):e214562. doi: 10.1001/jamahealthforum.2021.4562 (PMC8903104; doi:10.1001/jamahealthforum.2021.4562)
Supplement: Supplement. — eMethods. Propensity Score Weight Adjustment eTable 1. List of Covariates Used in Estimation of Propensity Score Adjustment eTable 2. Hosmer-Lemeshow Goodness of Fit Test for Propensity Score eFigure. Sample Selection Flowchart eTable 3. Comparing Medicare Beneficiaries with Disabilities Included Versus Excluded From Study Sample, Ages 18-64, 2015-2018 eTable 4. Characteristics of Community-Dwelling Medicare Beneficiaries with Disability Entitlement Enrolled in Medicare Advantage Versus Traditional Medicare Insurance, Ages 18-64, 2015-2018. After Relaxing Continuous Enrollment Inclusion Criteria eTable 5. Characteristics of Community-Dwelling Medicare Advantage Beneficiaries with Disability Entitlement by Special Needs Plan vs Non Special Needs Plan Enrollment, Ages 18-64, 2015-2018 eTable 6. Association of Medicare Advantage vs. Traditional Medicare with Ambulatory Care Access and Quality for Beneficiaries with Disability Entitlement, 2015-2018. With Dartmouth Hospital Referral Region Fixed Effects eTable 7. Association of Medicare Advantage vs. Traditional Medicare with Ambulatory Care Access and Quality for Beneficiaries with Disability Entitlement, 2015-2018. After Relaxing Continuous Enrollment Inclusion Criteria eTable 8. Association of Medicare Advantage vs. Traditional Medicare with Ambulatory Care Access and Quality for Beneficiaries with Disability Entitlement, 2015-2018. Testing for Heterogeneous Treatment Effects by Level of Functional Impairment [file jamahealthforum-e214562-s001.pdf]

Supplemental Online Content

Johnston KJ, Wen H, Pollack HA. Comparison of ambulatory care access and quality for beneficiaries with disabilities covered by Medicare Advantage vs traditional Medicare insurance. *JAMA Health Forum.* 2022;3(1):e214562. doi:10.1001/jamahealthforum.2021.4562

eMethods. Propensity Score Weight Adjustment.

eTable 1. List of Covariates Used in Estimation of Propensity Score Adjustment.

eTable 2. Hosmer-Lemeshow Goodness of Fit Test for Propensity Score.

eFigure. Sample Selection Flowchart

eTable 3. Comparing Medicare Beneficiaries with Disabilities Included Versus Excluded From Study Sample, Ages 18-64, 2015-2018

eTable 4. Characteristics of Community-Dwelling Medicare Beneficiaries with Disability Entitlement Enrolled in Medicare Advantage Versus Traditional Medicare Insurance, Ages 18-64, 2015-2018. After Relaxing Continuous Enrollment Inclusion Criteria

eTable 5. Characteristics of Community-Dwelling Medicare Advantage Beneficiaries with Disability Entitlement by Special Needs Plan vs Non Special Needs Plan Enrollment, Ages 18-64, 2015-2018

eTable 6. Association of Medicare Advantage vs. Traditional Medicare with Ambulatory Care Access and Quality for Beneficiaries with Disability Entitlement, 2015-2018. With Dartmouth Hospital Referral Region Fixed Effects

eTable 7. Association of Medicare Advantage vs. Traditional Medicare with Ambulatory Care Access and Quality for Beneficiaries with Disability Entitlement, 2015-2018. After Relaxing Continuous Enrollment Inclusion Criteria

eTable 8. Association of Medicare Advantage vs. Traditional Medicare with Ambulatory Care Access and Quality for Beneficiaries with Disability Entitlement, 2015-2018. Testing for Heterogeneous Treatment Effects by Level of Functional Impairment

eReferences

© 2022 Johnston KJ et al. *JAMA Health Forum.*

eMethods. Propensity Score Weight Adjustment.

Following the approach previously described by Garrido et al.[1] and similar to the approach previously implemented in the Medicare Current Beneficiary Survey (MCBS) by Cutler et al.,[2,3] the entire sample was reweighted by propensity to enroll in Medicare Advantage (MA) as predicted by observed confounders. Specifically, the entire sample of beneficiaries with disabilities was reweighted by the inverse probability of treatment, defined as enrollment in MA vs traditional Medicare (TM). This was done by calculating the inverse of normalized propensity score weights that were created by estimating a multivariable logistic regression model at the person-year level with the beneficiary demographic, insurance, social, health, and local area covariates listed in **eTable 1** used to predict MA (vs TM) enrollment. The resultant inverse probability of treatment weights were multiplied by the MCBS weights to make them nationally representative. The propensity-adjusted results thus change the distribution of confounders in both MA and TM beneficiaries so that they are the same as the distribution in the entire nationally representative sample, and then by multiplying them with the MCBS weights, the same as the distribution in the national population of beneficiaries with disability entitlement. Model fit was assessed by the Hosmer-Lemeshow test and balance of covariates assessed by comparing reweighted descriptive statistics on each of the covariates for MA vs TM beneficiaries, using the Wald test to assess whether there remained any significant differences. The Hosmer-Lemeshow test results on model fit for the regression model used to calculate propensity score weights indicate a good fit (p=0.74, **eTable 2**). Covariates were balanced after reweighting the sample with no remaining significant (p<.05) differences on any beneficiary characteristics for MA vs TM beneficiaries (**Main Table 2**). In addition, 99.1% of sample observations were on the area of common support for propensity scores.

© 2022 Johnston KJ et al. *JAMA Health Forum.*

eTable 1. List of Covariates Used in Estimation of Propensity Score Adjustment.

[1]	Age - continuous	[18]	Obese
[2]	Age Squared - continuous	[19]	Poor Self-rated Health
[3]	Male	[20]	ADLs with difficulty/can't do - continuous 0-6
[4]	Black	[21]	IADLs with difficulty/can't do - continuous 0-6
[5]	Hispanic	[22]	Diabetes
[6]	White	[23]	Heart Failure
[7]	Veteran	[24]	Ischemic Heart Disease
[8]	Medicaid	[25]	COPD/Asthma
[9]	Private Insurance	[26]	Mental Health Condition
[10]	Part D	[27]	Intellectual/Developmental Disability
[11]	Income - continuous	[28]	Rural
[12]	Poverty Status ≤ 100% FPL	[29]	MA Market Penetration Rate - continuous
[13]	Highschool Graduate	[30]	Medicaid * Hispanic
[14]	College Graduate	[31]	Medicaid * Male
[15]	Lives Alone	[32]	Part D * MA Market Penetration Rate
[16]	Current Smoker	[33]	Private Insurance * Poverty Status
[17]	Alcohol Abuse	[34]	MCBS Survey Weight - continuous

Notes: Poverty Status is income ≤100% federal poverty level; ADLs is Activities of Daily Living; IADLs is instrumental ADLs; MA is Medicare Advantage; MCBS survey weight is the cross-sectional weight assigned to the individual beneficiary-year observation to make it nationally representative and to account for the overall annual selection probability of each person sampled and include adjustments for the stratified sampling design, survey nonresponse, and coverage error.

© 2022 Johnston KJ et al. *JAMA Health Forum.*

eTable 2. Hosmer-Lemeshow Goodness of Fit Test for Propensity Score.

Group	Medicare Advantage Enrollment				Traditional Medicare Enrollment			
	Probability	Observed	Expected		Observed	Expected		Total
**1**	0.0792	37	32.6		684	688.4		721
**2**	0.1381	84	79.7		636	640.3		720
**3**	0.1913	111	119		609	601		720
**4**	0.2480	153	157.3		567	562.7		720
**5**	0.3123	204	201.3		516	518.7		720
**6**	0.3785	228	248.4		492	471.6		720
**7**	0.4499	303	297.3		417	422.7		720
**8**	0.5302	355	352.1		365	367.9		720
**9**	0.6488	433	422.9		287	297.1		720
**10**	0.9479	536	533.3		184	186.7		720
Number of Observations =					7201			
Number of Groups =					10			
Hosmer-Lemeshow χ2 statistic (8 DF) =					5.14			
Prob > χ2 =					0.742			

Notes: Probability is the propensity score calculated from the multivariable logistic regression model predicting Medicare Advantage enrollment using the covariates listed in eTable 1. DF is degrees of freedom.

© 2022 Johnston KJ et al. *JAMA Health Forum.*

eFigure. Sample Selection Flowchart.

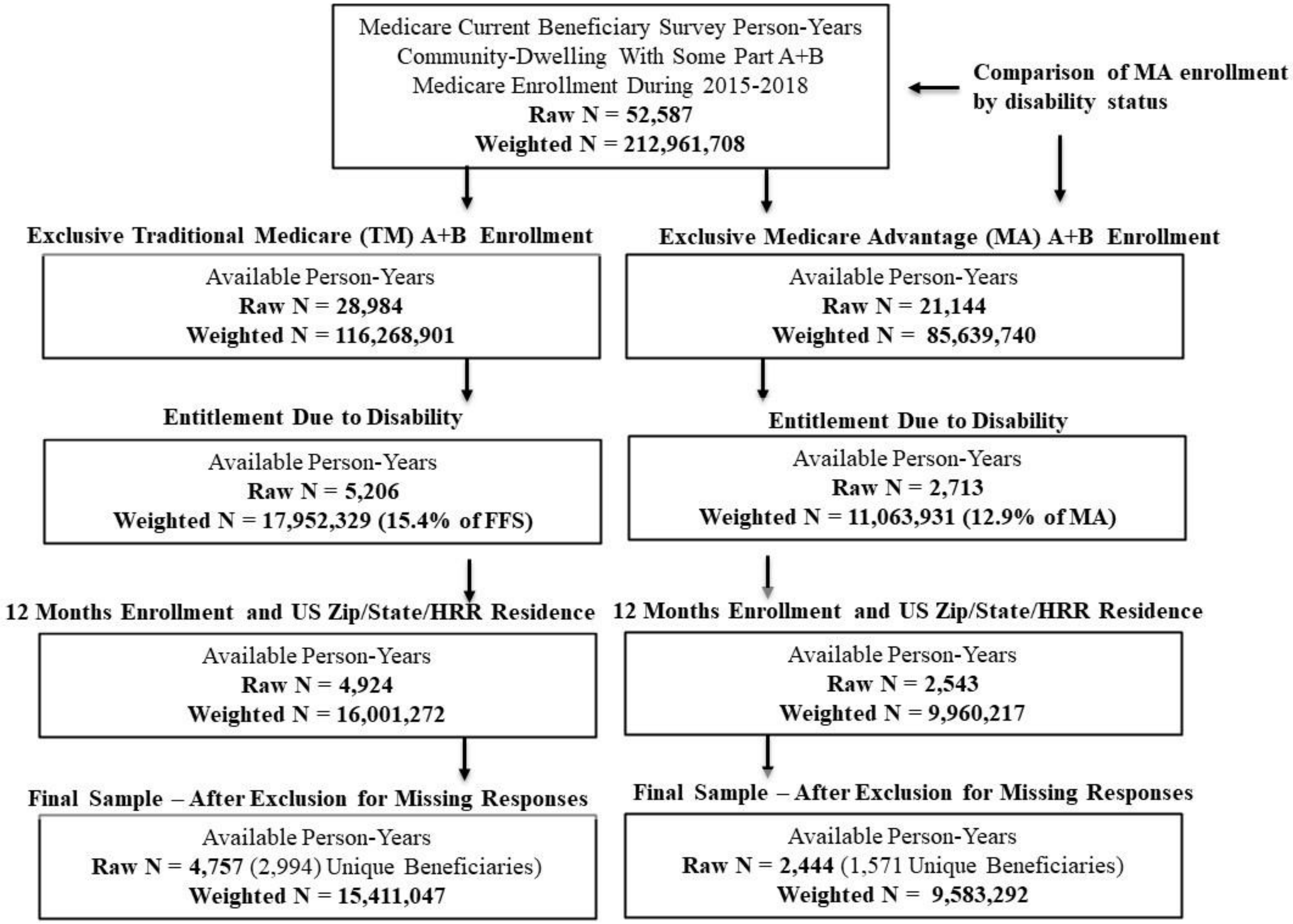

© 2022 Johnston KJ et al. *JAMA Health Forum.*

eTable 3. Comparing Medicare Beneficiaries with Disabilities Included Versus Excluded From Study Sample, Ages 18-64, 2015-2018.

	Study Sample Beneficiaries[a]	Excluded Beneficiaries[b]		P-Value[c]
**Total Number of Patient Years, Unweighted, N[d]**	**7,201**		**718**	**--**
**Sample Selection Characteristics**				
Medicare Advantage Enrollment	38.3%	718	36.8%	0.58
Traditional Medicare Enrollment	61.7%		63.2%	
Months Enrolled, *MEAN*	12.0		8.5	<.001
Died During Year	0.2%		13.1%	<.001
**Demographic Characteristics**				
Age in Years, *MEAN (SD)*	52.1 (11.0)	718	52.2 (9.3)	0.81
Ages 18-24	1.7%		3.8%	<.001
Ages 25-29	3.0%		3.8%	0.20
Ages 30-34	4.8%		3.0%	0.005
Ages 35-39	5.6%		4.2%	0.05
Ages 40-44	7.2%		6.2%	0.23
Ages 45-49	9.0%		7.8%	0.46
Ages 50-54	17.6%		16.7%	0.71
Ages 55-59	26.1%		26.3%	0.95
Ages 60-64	25.0%		28.2%	0.21
Sex				
Male	50.5%		51.6%	0.73
Female	49.5%		48.4%	
Minority Race and Ethnicity	30.7%		35.6%	0.001
Black	17.4%		14.5%	
Hispanic	10.2%		17.0%	
Native American	1.4%		2.0%	
Asian/Pacific Islander	1.6%		2.1%	
Other Race and Ethnicity	69.3%		59.4%	
White	65.1%		54.5%	
Multiracial	4.2%		4.9%	
Veteran (served in U.S. Armed Forces)	7.7%		7.3%	0.75
**Other Health Insurance**				
Medicaid (dually-enrolled)	54.0%	718	46.8%	0.02
Private (including medical, drug, vision, and dental)	20.0%		25.6%	0.02
Medicare Part D (standalone or with Part C)	87.0%		81.4%	0.006
**Social Risk Factors**				
Annual Income in Thousands, *MEAN (SD)*	25.3 (34.1)	718	28.7 (31.4)	0.15
Poverty (≤100% of Federal Poverty Level)	41.4%		36.1%	0.08

© 2022 Johnston KJ et al. *JAMA Health Forum.*

Education				
No High School or College Education	21.9%		26.2%	0.25
High School / Some College Education	69.3%		66.0%	
College / Graduate School Education	8.8%		7.8%	
Lives Alone	28.4%	585	22.8%	0.06
**Health Behaviors and Status**				
Current Smoker	32.8%	416	25.0%	0.02
Alcohol Abuse (≥4 Alcoholic Drinks Most Days)	19.7%		15.6%	0.16
Obese (Body Mass Index ≥30)	46.1%		50.2%	0.25
Poor Self-Rated Health	56.4%		56.9%	0.90
ADLS with Difficulty/Can't Do (0-6), *MEAN (SD)*	1.4 (1.8)		1.6 (1.3)	0.18
IADLs with Difficulty/Can't Do (0-6), *MEAN (SD)*	1.8 (1.8)		1.9 (1.3)	0.54
**Health Conditions**				
Diabetes	36.8%	491	36.8%	0.99
Heart Failure	9.1%		9.5%	0.84
Ischemic Heart Disease	15.9%		17.8%	0.46
COPD/Asthma	32.5%		31.5%	0.76
Mental Health Condition[e]	65.0%	718	44.3%	<.001
Intellectual and/or Developmental Disability	15.4%		8.9%	<.001
**Local Area Characteristics**				
Rural	24.4%	644	20.9%	0.18
Urban	75.6%		79.1%	
Medicare Advantage Market Penetration Rate	32.5%	715	36.2%	0.001
**Ambulatory Care Access (Study N=6,525)**				
Usual Source of Care	87.0%	309	81.6%	0.10
Usual Source of Care is PCP	73.0%		67.8%	0.18
Specialist Visit	48.1%		46.7%	0.75
**Ambulatory Care Quality**				
Blood Cholesterol Checked in Past Year[f] (Study N=2,715)	88.4%	149	84.2%	0.53
Annual Flu Shot (Study N=6,462)	55.4%	511	48.5%	0.06
Colon Cancer Screening[g] (Study N=3,233)	60.4%	186	62.2%	0.44

Abbreviations: N, number; ADLs, activities of daily living; IADLs, instrumental ADLs; COPD, chronic obstructive pulmonary disease; PCP, primary care clinician; MCBS, Medicare Current Beneficiary Survey.

[a]Medicare study sample beneficiaries ages 18-64 living in the community in a U.S. zip code and Hospital Referral Region with a current entitlement status of disabled, with at least 1-calendar-year excusive continuous enrollment in Medicare and completed the annual survey round in the MCBS.

[b]The excluded sample are Medicare beneficiaries ages 18-64 living in the community with a current entitlement status of disabled and at least 1 month of exclusive enrollment in Medicare.

[c]P-value on the Wald Test of significance, equivalent to the F-statistic for continuous variables and the Chi-squared statistic for categorical variables.

© 2022 Johnston KJ et al. *JAMA Health Forum.*

[d]Reporting unweighted sample size. Estimates from the 2015-2018 MCBS weighted to be nationally representative using cross-sectional weights accounting for the overall annual selection probability of each person sampled and including adjustments for the stratified sampling design, survey nonresponse, and coverage error.	
[e]Self-reported any psychiatric illness, including depression.	
[f]Self-reported having diabetes, ischemic heart disease, or heart failure and responded to MCBS questions for outcome variable	
[g]Fecal occult blood test at home or doctor's office or colonoscopy or sigmoidoscopy within past 5 years, excluding patients who self-reported having colon cancer or are under age 45.	

© 2022 Johnston KJ et al. *JAMA Health Forum.*

eTable 4. Characteristics of Community-Dwelling Medicare Beneficiaries with Disability Entitlement Enrolled in Medicare Advantage Versus Traditional Medicare Insurance, Ages 18-64, 2015-2018. After Relaxing Continuous Enrollment Inclusion Criteria.

	Medicare Advantage[a]		Traditional Medicare[b]		P-Value[c]
**Total Number of Patient Years, Unweighted, N[d]**		**2,643**		**5,201**	**--**
**Sample Selection Characteristics**	**N=**		**N=**		
Months Enrolled, *MEAN*	2643	11.6	5201	11.5	0.05
Died During Year		2.1%		1.9%	0.60
**Demographic Characteristics**					
Age in Years, *MEAN (SD)*	2643	54.1 (9.0)	5201	50.8 (11.7)	<.001
Sex					
Male		48.6%		51.9%	0.07
Female		51.4%		48.1%	
Minority Race and Ethnicity		35.9%		27.8%	<.001
Black		19.2%		15.9%	
Hispanic		14.3%		8.2%	
Native American		1.4%		1.6%	
Asian/Pacific Islander		0.9%		2.1%	
Other Race and Ethnicity		63.5%		71.5%	
White		58.9%		67.3%	
Multiracial		4.6%		4.1%	
Veteran (served in U.S. Armed Forces)		6.5%		8.5%	0.08
**Other Health Insurance**					
Medicaid (dually-enrolled)	2643	49.8%	5201	55.6%	0.02
Private (including medical, drug, vision, and dental)		13.4%		25.3%	<.001
Medicare Part D (standalone or with Part C)		96.9%		79.7%	<.001
**Social Risk Factors**					
Annual Income in Thousands, *MEAN (SD)*	2643	26.1 (37.6)	5201	25.6 (31.2)	0.76
Poverty (≤100% of Federal Poverty Level)		36.7%		42.8%	0.002
Education					
No High School or College Education		21.7%		21.7%	0.11
High School / Some College Education		68.1%		68.1%	
College / Graduate School Education		10.2%		10.2%	
Lives Alone	2593	29.7%	5118	29.7%	0.12
**Health Behaviors and Status**					

© 2022 Johnston KJ et al. *JAMA Health Forum.*

Current Smoker	2537	31.1%	5006	33.0%	0.37
Alcohol Abuse (≥4 Alcoholic Drinks Most Days)		20.4%		18.7%	0.29
Obese (Body Mass Index ≥30)		45.7%		47.1%	0.51
Poor Self-Rated Health		56.2%		56.3%	0.96
ADLS with Difficulty/Can't Do (0-6), *MEAN (SD)*		1.4 (1.7)		1.4 (1.9)	0.87
IADLs with Difficulty/Can't Do (0-6), *MEAN (SD)*		1.7 (1.6)		1.9 (1.9)	0.02
**Health Conditions**					
Diabetes	2562	40.5%	5056	34.5%	0.003
Heart Failure		9.7%		9.0%	0.55
Ischemic Heart Disease		18.5%		14.6%	0.01
COPD/Asthma		34.0%		31.4%	0.15
Mental Health Condition[e]	2643	60.6%	5201	62.6%	0.30
Intellectual and/or Developmental Disability		13.1%		15.3%	0.07
**Local Area Characteristics**					
Rural	2641	17.1%	5200	28.0%	<.001
Urban		82.9%		72.0%	
Medicare Advantage Market Penetration Rate	2642	37.7%	5201	29.7%	<.001

Abbreviations: N, number; ADLs, activities of daily living; IADLs, instrumental ADLs; COPD, chronic obstructive pulmonary disease; MCBS, Medicare Current Beneficiary Survey.

[a]Medicare Advantage beneficiaries ages 18-64 living in the community in a U.S. zip code with a current entitlement status of disabled, at least 1-month of enrollment in Part A+B benefits, and at least one MCBS survey completed.

[b]Traditional Medicare beneficiaries ages 18-64 living in the community in a U.S. zip code with a current entitlement status of disabled, at least 1-month of enrollment in Part A+B benefits, and at least one MCBS survey completed.

[c]P-value on the Wald Test of significance, equivalent to the F-statistic for continuous variables and the Chi-squared statistic for categorical variables.

[d]Reporting unweighted sample size. Estimates from the 2015-2018 MCBS weighted to be nationally representative using cross-sectional weights accounting for the overall annual selection probability of each person sampled and including adjustments for the stratified sampling design, survey nonresponse, and coverage error.

[e]Self-reported any psychiatric illness, including depression.

© 2022 Johnston KJ et al. *JAMA Health Forum.*

eTable 5. Characteristics of Community-Dwelling Medicare Advantage Beneficiaries with Disability Entitlement by Special Needs Plan vs Non Special Needs Plan Enrollment, Ages 18-64, 2015-2018.

	**Special Needs Plan**	**Non Special Needs Plan[a]**	**P-Value[b]**
**Total Number of Patient Years, Unweighted, N[c]**	**762**	**1,399**	**--**
**Demographic Characteristics**			
Age in Years, *MEAN (SD)*	51.3 (11.3)	55.5 (7.4)	<.001
Sex			
Male	44.0%	48.4%	0.30
Female	56.0%	51.6%	
Minority Race and Ethnicity	48.1%	28.8%	0.004
Black	25.9%	15.4%	
Hispanic	20.3%	11.4%	
Native American	1.0%	1.2%	
Asian/Pacific Islander	0.9%	0.8%	
Other Race and Ethnicity	51.9%	71.2%	
White	49.7%	65.4%	
Multiracial	2.2%	5.8%	
Veteran (served in U.S. Armed Forces)	2.5%	8.0%	0.03
**Other Health Insurance**			
Medicaid (dually-enrolled)	95.7%	32.9%	<.001
Private (including medical, drug, vision, and dental)	6.5%	12.4%	0.007
Medicare Part D (standalone or with Part C)	100.0%	98.8%	NE
**Social Risk Factors**			
Annual Income in Thousands, *MEAN (SD)*	17.6 (54.6)	27.4 (28.1)	<.001
Poverty (≤100% of Federal Poverty Level)	62.9%	27.3%	<.001
Education			
No High School or College Education	34.8%	17.0%	<.001
High School / Some College Education	62.2%	70.7%	
College / Graduate School Education	3.0%	12.3%	
Lives Alone	31.9%	29.3%	0.49
**Health Behaviors and Status**			
Current Smoker	38.4%	30.7%	0.04
Alcohol Abuse (≥4 Alcoholic Drinks Most Days)	20.8%	21.6%	0.78
Obese (Body Mass Index ≥30)	47.4%	44.8%	0.47
Poor Self-Rated Health	59.0%	57.7%	0.74
ADLS with Difficulty/Can't Do (0-6), *MEAN (SD)*	1.5 (2.0)	1.5 (1.6)	0.91
IADLs with Difficulty/Can't Do (0-6), *MEAN (SD)*	1.8 (1.9)	1.7 (1.5)	0.63

© 2022 Johnston KJ et al. *JAMA Health Forum.*

**Health Conditions**			
Diabetes	47.3%	38.8%	0.01
Heart Failure	11.8%	9.8%	0.46
Ischemic Heart Disease	22.0%	17.7%	0.21
COPD/Asthma	38.7%	33.2%	0.23
Mental Health Condition[d]	62.8%	64.6%	0.64
Intellectual and/or Developmental Disability	17.0%	11.5%	0.02
**Local Area Characteristics**			
Rural	18.4%	16.7%	0.75
Urban	81.6%	83.3%	
Medicare Advantage Market Penetration Rate	39.6%	37.4%	0.12

Abbreviations: N, number; ADLs, activities of daily living; IADLs, instrumental ADLs; COPD, chronic obstructive pulmonary disease; MCBS, Medicare Current Beneficiary Survey; NE, not estimable.

[a]Special Needs Plans (SNPs) for chronic and disabling conditions, dually enrolled Medicare-Medicaid beneficiaries, and beneficiaries institutionalized in long-term care.

[b]P-value on the Wald Test of significance, equivalent to the F-statistic for continuous variables and the Chi-squared statistic for categorical variables.

[c]Reporting unweighted sample size for Medicare Advantage beneficiaries with available plan and contract data in the MCBS ages 18-64 living in the community in a U.S. zip code with a current entitlement status of disabled, at least 1-calendar-year of continuous enrollment in Part A+B benefits, and completed the annual MCBS survey rounds. Estimates from the 2015-2018 MCBS weighted to be nationally representative using cross-sectional weights accounting for the overall annual selection probability of each person sampled and including adjustments for the stratified sampling design, survey nonresponse, and coverage error.

[d]Mental health conditions include any self-reported psychiatric illness, including depression

© 2022 Johnston KJ et al. *JAMA Health Forum.*

eTable 6. Association of Medicare Advantage vs. Traditional Medicare with Ambulatory Care Access and Quality for Beneficiaries with Disability Entitlement, 2015-2018. With Dartmouth Hospital Referral Region Fixed Effects.

		Adjusted Marginal Difference of MA vs TM	
		**Regression Results (95% CI)[a]**	**Propensity-Weighted Regression Results (95% CI)[b]**
**Access[c]**			
Usual Source of Care, %		2.0 (0.7, 3.2)	1.4 (0.0, 2.8)
Usual Source of Care is PCP, %		4.6 (0.7, 8.4)	2.6 (-1.1, 6.4)
Specialist Visit, %		5.4 (1.3, 9.4)	6.1 (1.6, 10.7)
**Quality**			
Annual Cholesterol Screen, %[d]		0.9 (0.0, 1.8)	1.3 (0.2, 2.4)
Annual Flu Shot, %[e]		11.3 (6.7, 15.9)	11.8 (6.8, 16.8)
Colon Cancer Screening, %[f]		10.4 (5.1, 15.7)	10.2 (4.6, 15.9)

Abbreviations: CI, confidence interval; PCP, primary care provider; MA, Medicare Advantage.

[a]We estimated multivariable logistic regression models for each outcome that also adjusted for the characteristics listed in Table 2 (with race/ethnicity collapsed into minority vs. other). We added fixed effects for the Dartmouth hospital referral regions that beneficiaries resided in to control for regional differences in supply of medical services, clinician practice intensity, and coding intensity. We included year fixed effects to control for secular trend and adjusted our p-values for the complex survey design of the MCBS and intra-person correlation over time. We used Stata's Margins command to report our results as the marginal difference of MA vs. TM for the dependent variables by modeling the response in the dependent variables to the exposure variable at the population means.

[b]We estimated the same multivariable logistic regression models as in a, but this time reweighting the sample using the propensity score weights described previously in order to change the distribution of observed confounders in both the treated (Medicare Advantage) and untreated (traditional Medicare) beneficiaries so that they are the same as the distribution in the entire sample. These estimates should be interpreted as what we would expect to see if every Medicare beneficiary in our nationally representative sample enrolled in Medicare Advantage vs what we would expect to see if no-one enrolled in Medicare Advantage (i.e. the average treatment effects).

[c]Unweighted sample n=6,525. Met baseline study inclusion and responded to MCBS questions for outcome variables.

© 2022 Johnston KJ et al. *JAMA Health Forum.*

[d]Unweighted sample n=2,715. Met baseline study inclusion and exclusion criteria and self-reported having diabetes, ischemic heart disease, or heart failure and responded to MCBS questions for outcome variable.
[e]Unweighted sample n=6,462. Met baseline study inclusion and exclusion criteria and responded to MCBS question for outcome variable.
[f]Fecal occult blood test at home or doctor's office or colonoscopy or sigmoidoscopy within past 5 years, excluding patients who self-reported having colon cancer or are under age 45. Unweighted sample n=3,233 for patients who met above criteria as well as baseline study inclusion and exclusion criteria and responded to MCBS questions for outcome variable.

© 2022 Johnston KJ et al. *JAMA Health Forum.*

eTable 7. Association of Medicare Advantage vs. Traditional Medicare with Ambulatory Care Access and Quality for Beneficiaries with Disability Entitlement, 2015-2018. After Relaxing Continuous Enrollment Inclusion Criteria.

		Unadjusted Results			Adjusted Marginal Difference
		**Medicare Advantage**	**Traditional Medicare**	**Absolute Difference (95% CI)**	**Medicare Advantage (95% CI)[a]**
**Access[b]**					
Usual Source of Care, %		90.3	84.5	5.8 (3.7, 8.0)	4.2 (1.8, 6.6)
Usual Source of Care is PCP, %		77.5	69.6	7.9 (4.1, 11.7)	5.3 (1.4, 9.3)
Specialist Visit, %		53.3	44.4	8.9 (5.0, 12.8)	4.5 (0.8, 8.3)
**Quality**					
Annual Cholesterol Screen, %[c]		90.8	85.9	5.0 (1.8, 8.2)	4.3 (1.1, 7.5)
Annual Flu Shot, %[d]		61.9	50.4	11.5 (7.4, 15.5)	11.5 (6.7, 16.4)
Colon Cancer Screening, %[e]		68.4	54.7	13.7 (9.5, 18.0)	11.3 (6.8, 15.9)

Abbreviations: CI, confidence interval; PCP, primary care clinician; MA, Medicare Advantage.

[a]We estimated multivariable logistic regression models for each outcome that also adjusted for the demographic, health insurance, social risk, and local are characteristics listed in Table 2 (with race/ethnicity collapsed into minority vs. other and excluding living alone). We added fixed effects for the states that beneficiaries resided in to control for state policy differences and state differences in supply of medical services, clinician practice intensity, and coding intensity. We included year fixed effects to control for secular trend and adjusted our p-values for the complex survey design of the MCBS and intra-person correlation over time. We used Stata's Margins command to report our results as the marginal difference of MA vs. TM for the dependent variables by modeling the response in the dependent variables to the exposure variable at the population means.

[b]Unweighted sample n=6,760 (unadjusted) and 6,725 (adjusted). Met baseline study inclusion and responded to MCBS questions for outcome variables.

[c]Unweighted sample n=2,837 (unadjusted) and 2,815 (adjusted). Met baseline study inclusion and exclusion criteria and self-reported having diabetes, ischemic heart disease, or heart failure and responded to MCBS questions for outcome variable.

[d]Unweighted sample n=6,901 (unadjusted) and 6,865 (adjusted). Met baseline study inclusion and exclusion criteria and responded to MCBS question for outcome variable.

[e]Fecal occult blood test at home or doctor's office or colonoscopy or sigmoidoscopy within past 5 years, excluding patients who self-reported having colon cancer or are under age 45. Unweighted sample n=3,380 (unadjusted) and 3,355 (adjusted) for patients who met above criteria as well as baseline study inclusion and exclusion criteria and responded to MCBS questions for outcome variable.

© 2022 Johnston KJ et al. *JAMA Health Forum.*

eTable 8. Association of Medicare Advantage vs. Traditional Medicare with Ambulatory Care Access and Quality for Beneficiaries with Disability Entitlement, 2015-2018. Testing for Heterogeneous Treatment Effects by Level of Functional Impairment.

		Adjusted Marginal Difference[a]		
		**Medicare Advantage (95% CI)**	**Interaction of Medicare Advantage & Count of ADL Limitations (95% CI)**	**Interaction of Medicare Advantage & Count of IADL Limitations (95% CI)**
**Access[b]**				
Usual Source of Care, %		5.1 (1.7, 8.4)	0.4 (-1.1, 2.0)	-1.3 (-2.8, 0.2)
Usual Source of Care is PCP, %		6.3 (1.0, 11.5)	-0.8 (-3.2, 1.6)	-0.1 (-2.3, 2.1)
Specialist Visit, %		7.4 (1.4, 13.4)	2.9 (0.3, 5.5)	-3.9 (-6.6, -1.1)
**Quality**				
Annual Cholesterol Screen, %[c]		2.9 (-0.6, 6.5)	1.2 (-0.4, 2.8)	-0.8 (-2.7, 1.2)
Annual Flu Shot, %[d]		8.5 (2.1, 14.8)	-0.6 (-3.4, 2.1)	1.5 (-1.5, 4.4)
Colon Cancer Screening, %[e]		12.9 (5.6, 20.2)	2.2 (-0.9, 5.4)	-2.8 (-5.9, 0.3)

Abbreviations: CI, confidence interval; PCP, primary care clinician; MA, Medicare Advantage.

[a]We estimated multivariable logistic regression models for each outcome that also adjusted for the characteristics listed in Table 2 (with race/ethnicity collapsed into minority vs. other and with functional impairment defined as below instead of count of ADL and IADL limitations). We added fixed effects for the state that beneficiaries resided in to control for regional differences in supply of medical services, clinician practice intensity, and coding intensity. We included year fixed effects to control for secular trend and adjusted our p-values for the complex survey design of the MCBS and intra-person correlation over time. We used Stata's Margins command to report our results as the marginal difference of MA vs. TM, and the interaction of MA with beneficiaries' counts of ADL and IADL limitations, respectively, on the dependent variables by modeling the response in the dependent variables to these exposure variables at the population means.

[b]Unweighted sample n=6,525. Met baseline study inclusion and responded to MCBS questions for outcome variables.

© 2022 Johnston KJ et al. *JAMA Health Forum.*

[c]Unweighted sample n=2,715. Met baseline study inclusion and exclusion criteria and self-reported having diabetes, ischemic heart disease, or heart failure and responded to MCBS questions for outcome variable.
[d]Unweighted sample n=6,462. Met baseline study inclusion and exclusion criteria and responded to MCBS question for outcome variable.
[e]Fecal occult blood test at home or doctor's office or colonoscopy or sigmoidoscopy within past 5 years, excluding patients who self-reported having colon cancer or are under age 45. Unweighted sample n=3,233 for patients who met above criteria as well as baseline study inclusion and exclusion criteria and responded to MCBS questions for outcome variable.

© 2022 Johnston KJ et al. *JAMA Health Forum.*

eReferences

1. Garrido MM, Kelley AS, Paris J, et al. Methods for Constructing and Assessing Propensity Scores. *Health Serv Res*. 2014;49(5):1701-1720. doi:10.1111/1475-6773.12182

2. Cutler DM, Ghosh K, Messer KL, Raghunathan TE, Stewart ST, Rosen AB. Explaining The Slowdown In Medical Spending Growth Among The Elderly, 1999–2012. *Health Aff (Millwood)*. 2019;38(2):222-229. doi:10.1377/hlthaff.2018.05372

3. Cutler D, Ghosh K, Messer K, Raghunathan T, Rosen A, Stewart S. *A Satellite Account for Health in the United States*. National Bureau of Economic Research; 2020:w27848. doi:10.3386/w27848

© 2022 Johnston KJ et al. *JAMA Health Forum.*
